# Hepatitis B vaccination in Nepalese infants: The present scenario

**DOI:** 10.3126/nje.v7i3.19007

**Published:** 2017-09-30

**Authors:** Sudhir Adhikari, Brijesh Sathian

**Affiliations:** 1 Assistant Professor, Department of Pediatrics, Manipal College of Medical Sciences Pokhara, Nepal.; 2 Academic Research Associate, Trauma Surgery Hamad General Hospital, P. O BOX 3050 Doha, Qatar

According to the latest WHO 2015 estimation, 257 million people were infected with hepatitis B virus and a mortality of 

 887000 (because of associated hepatocellular carcinoma and cirrhosis). In addition, prevalence of Hepatitis B in WHO South-East Asia Region was 2.0% [ 1 ]. Infections acquired during infancy and young age carry a high risk of evolution into chronic HBsAg carrier stage. 

 Preventive strategies along with Hepatitis B vaccine have a great impact to reduce the incidence of new infections over last few decades. Passive immunization with hepatitis B immune globulin (HBIG) and active immunization with Hepatitis B vaccine has resulted in a sudden decline in the disease incidence. Immunization of infants significantly reduces the incidence of vertical transmission of the virus from the affected mothers. This is probably the most effective health intervention. 

 The government of Nepal along with the aid of GAVI had introduced Hepatitis B vaccine in phase-wise manner from 2002 to 2004. It is currently administered as penta-valent vaccine along with Hemophilus influenza B(HiB) and Diphtheria Pertussis and Tetanus(DPT) vaccines at 6, 10 and 14 weeks of age [ 2 ]. Infants those who are born from hepatitis B surface antigen (HBsAg) negative mothers can effectively be protected from the hepatitis B viral infections by good immunization coverage. 

However, infants born with HBsAg positive mothers need national strategies for active as well as passive immunization. Hepatitis B vaccine is recommended within 24 hours of life by the American academy of pediatrics (AAP). Additional 0.5 mL of HBIG within 12 hours of birth is indicated for infants of mothers with HBsAg+ or unknown HBsAg status [ 3 ]. HBIG should be administered within 72 hours for maximum benefit but not useful beyond 7 days. HBsAg antibody titres should be done at age 9 to12 months or 1 to 2 months after completion of the HepB vaccination [ 3 ]. A systematic review done by Machaira et al reported that the effectiveness of vaccine alone compared with vaccine plus HBIG for the immunization of babies of HBsAg+/HBeAg- mothers were equal [ 4 ]. 

 In 2012, Upreti et al had reported a low burden of chronic hepatitis B viral infection among children born in both the pre(0.28%) and the post-vaccination (0.13%) era [ 5 ]. However, another study carried out in a remote District of Western Nepal in the year 2014 had shown high burden of hepatitis B infections in mothers and children younger than 5 years [ 6 ]. This raises the concern that HBsAg carrier rates can be higher than the national projection in marginalized and rural population. 

 There are few concerns about effective prevention of hepatitis B viral infection strategies at birth. A significant number of mothers do not have antenatal visit and home delivery is also common. There is lack of policy for administration of HBIG along with a birth dose of HBsAg vaccine to newborns born from HBsAg positive mothers. Regular availability of HBIG and HBsAg vaccine is another concern. Passive immunization also has additional financial burden on the families. 

 In the National Immunization Schedule (NIS) the Pentavalent vaccine (DPT, HiB and Hepatitis B) is given at 6, 10 and 14 weeks. There is a dilemma how to incorporate infants who receive birth dose of hepatitis B vaccine in this schedule. There is also lack of antibody titre measurement against HBsAg and follow up of these high-risk infants. A population- based study from southern Nepal had shown that though national coverage of routine immunization is reported to be high there is also a concern for delay and incomplete receipt of vaccines under NIS [ 7 ]. 

 Nepal has achieved a great progress in childhood immunization over decades. Health promotion and prevention has maintained the transmission of hepatitis B to a low rate in Nepal. But only 21 out of 75 districts in Nepal have achieved more than 90% DPT-Hep-Hib3 coverage in 2016 [ 8 ]. There is a noticeable 10% reduction in coverage from 2014 (92%) to 

 2016(81%) of DPT-Hep-Hib3 [ 8 ]. In addition, DPT-HepB- Hib vaccine wastage rate was greater than the accepted rate of 

 15% [ 8 ]. Under the sustainable Development Agenda 2030, WHO is supporting the countries for achieving the global hepatitis goals by formulating evidence-based policy and data for action, promoting partnerships and mobilizing resources, raising awareness, preventing transmission, and also improving the screening, care and treatment services [ 1 ]. There is an urgent need for a national policy for active and passive hepatitis B immunization to sustain our low transmission status.

**Abbreviations**

AAP- American academy of pediatrics 

 DPT- Diphtheria Pertussis and Tetanus 

 HBIG- hepatitis B immune globulin 

 HBsAg- hepatitis B surface antigen 

 HiB- Hemophilus influenza B 

 NIS- National immunization schedule

## References

[1] WHO. Hepatitis B. [online] 2017[cited 2017 May 23]. Available from: http://www.who.int/mediacentre/ factsheets/ fs204/en/

[2] Ministry of Health and Population. National Immunization Program. Reaching Every Child. National Immunization Program. Comprehensive Multi-Year Plan 2068-2072 (2011- 2016). [online] 2011; [cited 2017 May 1]. Available from: http://dohs.gov.np/wp-content/uploads/chd/ Immunization/ cMYP_2012_2016_May_2011.pdf

[3] CDC. Recommended Immunization Schedule for Children and Adolescents Aged 18 Years or Younger, United States. [online] 2017; [cited 2017 May 23]. Available from: https://www.aap.org/en- us/Documents/ immunizationschedule2017.pdf 10.15585/mmwr.mm6605e1 PMC5657960 28182607

[4] Machaira M Papaevangelou V Vouloumanou EK Tansarli GS Falagas ME. Hepatitis B vaccine alone or with hepatitis B immunoglobulin in neonates of HBsAg+/HBeAg- mothers: a systematic review and meta-analysis. J Antimicrob Chemother.2015 Feb; 70 (2): 396 - 404. https://doi.org/10.1093/jac/dku404 PMid: 25362571 10.1093/jac/dku404

[5] Upreti SR Gurung S Patel M Dixit SM Krause LK Shakya G Wannemuehler K Rajbhandari R Bohara R Schluter WW. Prevalence of chronic hepatitis B virus infection before and after implementation of a hepatitis B vaccination program among children in Nepal. Vaccine.2014 ; 32 (34): 4304 - 9. https://doi.org/10.1016/j.vaccine.2014.06.027 PMid: 24951865 10.1016/j.vaccine.2014.06.027 PMC4663719

[6] Shedain PR Devkota MD Banjara MR Ling H Dhital S Prevalence and risk factors of hepatitis B infection among mothers and children with hepatitis B infected mother in upper Dolpa, Nepal. BMC Infect Dis.2017 Oct; 17 (1): 667 . https://doi.org/10.1186/s12879-017-2763-4 PMCid: 29017456 10.1186/s12879-017-2763-4 PMC5633872

[7] Hughes MM Katz J Englund JA Khatry SK Shrestha L LeClerq SC Steinhoff M Tielsch JM. Infant vaccination timing: Beyond traditional coverage metrics for maximizing impact of vaccine programs, an example from southern Nepal. Vaccine.2016 ; 34 (7): 933 - 41. https://doi.org/10.1016/j.vaccine.2015.12.061 PMid: PMCid: 26788880 10.1016/j.vaccine.2015.12.061 PMC4744084

[8] Ministry of Health. Child Health. Annual Report 2017. [online] 2017; [cited 2017 May 23]. Available from: http://dohs.gov.np/wp-content/uploads/2017/06/ DoHS_Annual_Report_2072_73.pdf

